# A Country Health Officer and Board of Health1Presidential address delivered at the 80th annual meeting of the Medical Society of the County of Erie, January 8, 1901.

**Published:** 1901-02

**Authors:** E. H. Ballou

**Affiliations:** Gardenville, N. Y.


### A COUNTRY HEALTH OFFICER AND BOARD OF HEALTH.[1]

By E. H. BALLOU, M. D., Gardenville, N. Y.

WHAT I have to say in this brief paper in regard to health matters pertains to the Town of West Seneca, where I have held the position of health officer for the past fifteen years. This town joins the City of Buffalo on the south and east for over five miles, therefore all things along the sanitary line affecting the southern suburbs of Buffalo also affect the town. Our first board of health was organised in the year 1885, consisting of the Town Board, ex-officio, and a citizen whom they elect annually to act with them. The general duties of the Town Board of Health are: first, to elect a health officer and recorder of vital statistics, to have charge of records of births, deaths and marriages, to pass such laws and rules with penalties attached as they may see fit when not in conflict with state laws, to see that the health officer puts all rules in force, and to pass judgment upon all cases where there is an appeal from any decision of the health officer in regard to health matters. They have an annual meeting in March for organisation and special meeting at the call of the president of the board or health officer.

The first duty that devolved upon me as health officer was to take charge of a case of smallpox. I received word that a child at Limestone Hill was ill with symptoms of smallpox, and upon investigation I found it to be as reported. It was a Polish child, and its parents to eke out their meagre living kept boarders. The father with seven of the boarders was working on the railroad with a gang of over 100 men, so that the germs of the disease had had a great chance to spread. Prompt measures were taken to quarantine this case. Through the kindness of Father Baker of the reformatory, a cottage was procured in the woods, at least one quarter of a mile from any habitation and the inmates of the house were moved thence, with the exception of the boarders, who had disappeared. The house was kept under guard, provisions were brought to them, and at the termination of the case all bedding, clothing and furniture of every description was burned. Every man, woman and child at Limestone Hill was vaccinated and there was no spread of the disease.

One of the greatest evils that our board of health had to contend with was the spreading of garbage and night soil from the city upon

1. Presidential address delivered at the 80th annual meeting of the Medical Society of the County of Erie, January 8, 1901.

farms as fertilisers. This would not be objectionable were it done properly, that is, the fertiliser to be immediately ploughed under and allowed to mix with the soil; but this has not been the case and the result was one quite severe epidemic of typhoid fever. A large farm, about one mile from the city on Cazenovia Creek, was covered with night soil to the depth of two or three inches. This was exposed to rains and the heat of the sun for a number of weeks, the prevailing winds being southwesterly carried the sickening odor of night soil over a very thickly settled part of Seneca and adjoining streets. This odor was almost unbearable, so much so that the people were compelled to keep their doors and windows closed most of the time. Undoubtedly the germs of disease were also carried into this district, as within a short time typhoid fever appeared in nine different houses, and before a month had passed over thirty cases had been reported with many deaths. This to me was proof enough that garbage and night soil spread upon farms were unsanitary and not conducive to good health, and active and successful measures were taken to prevent the spread of illness and disease in this manner.

It has been our experience that quarantine and isolation have all to do with the spread of contagious disease. This has been exemplified time and time again; for instance, sporadic cases of scarlet fever have appeared in thickly settled portions of the town within fifty feet of a school house with no spread of the disease, whereas twenty years ago this dread disease entered our town from Lancaster and spread from school district to school district with over sixty deaths, this with out quarantine. Since that epidemic we have confined scarlet fever to only such cases as are contracted in some manner unknown, but generally upon a thorough investigation, we find that it comes from Buffalo. Whatever in regard to quarantine applies to scarlet fever applies also to diphtheria; that is, with the addition of the early use of antitoxin. There is not a doubt in my mind but that in time diphtheria can be entirely eradicated.

You all remember the epidemic of measles that spread through Buffalo last winter. Within a few days of its appearance in South Buffalo it followed out Seneca Street to school district number three, then to Ebenezer, from there to Reserve, and finally to Limestone Hill and Blasdell, closing all these schools from two to four weeks. Gardenville escaped, having had its experience about five years previous to that time.

The quarantine of measles I do not approve of for the following reasons: first, it is too late, for after a child is far enough advanced with measles to make a diagnosis of the case every one about it has been exposed for three or four days. Second, it keeps many well and healthy children out of school who expose no one to the disease excepting those who have been previously exposed. Third, this disease is very rarely fatal with children of school age.

With whooping cough my experience has been that all that is necessary is to isolate the case and not quarantine the entire family.

Immature or "bob veal," as it is commonly termed, caused us some trouble and expense. West Seneca was used as a dumping ground for all the bob veal in the south towns. It was brought in by the car load to the different depots and carted to Buffalo in the night by unscrupulous butchers, who disposed of it through the eastern part of the city, the Broadway market being made the distributing depot for most of it. The State Board of Health called our attention to the matter repeatedly, and we did what we could to prevent it, but it was a great expense, as we were obliged to station inspectors at all the depots for considerable length of time during the spring and fall. We therefore decided, if it were practicable, to shift this on to the city, as it did not interest us particularly, none of this veal having been offered for sale in our town. After consultation with Health Commissioner Wende, of Buffalo, I introduced the following resolution at a regular meeting of our board of health:

*Resolved*, That the Buffalo inspector of meats and provisions be and hereby is appointed inspector of meats and provisions for the Town of West Seneca, he to serve without pay or recompense of any kind, and for any claims for damages for the destruction of meats or provisions by him the Town of West Seneca will not hold itself responsible.

This resolution was passed upon and accepted by the attorney for the City of Buffalo, and at our next meeting it was unanimously adopted, thereby relieving us of a great deal of trouble and considerable expense and placing it upon the City of Buffalo, where it properly belonged. This seems to have been very acceptable to the city, also to the state board of health, for since that time the state board of health has given the board of health of Buffalo jurisdiction over a large territory adjoining it, as regards the seizure and destruction of bob veal.

At present we have no particular system of sewerage, the three large creeks passing through our town acting as trunk sewers. The Sulphur Springs Orphan Asylum discharges its sewage into the Buffalo Creek, as does the Sisters of St. Francis Home at Gardenville, but the reformatory at Limestone Hill, with nearly 1,000 inmates, has its principal sewerage system connected with a city trunk sewer. All of these institutions have their water from Buffalo. We have no use for an elaborate system of sewerage, but as the demand for such arises, there is no doubt but what our sewage will be taken care of by the city system, as will also that of the Steel Plant.

All of our water comes from either wells or cisterns, and is not of the best, as the most of it is tainted with iron, sulphur or oil. In the vicinity of Gardenville, wells cannot be over twenty feet deep, as below this the water is too salty for use. In the near future we expect to have as good, if not better, water than even the City of Buffalo, as the Depew and Lancaster Water Company, whose pipes traverse our town from end to end, has contracted to place their water wherever it is desired. This water is taken from Lake Erie, about two miles from the shore line at Stony Point, and this is so far above Buffalo that it will be impossible to be contaminated by city sewage, and as plenty of pure water is conducive to cleanliness and good health we expect it will be a great factor in the development of the town.

Hoping that these few rambling and disconnected remarks will give you some insight into the duties of a country health officer, I close by inviting you all to move out into the Town of West Seneca, where you are sure to be vaccinated when you have been exposed to the smallpox, quarantined when you have scarlet fever or diphtheria, *not* quarantined when you have the measles or whooping cough (if I can help it), watered and washed with pure water from Lake Erie and when you die, buried not on top of the ground as in Howard Cemetery in days gone by, but in a decent and orderly manner.

